# Mechanisms of coagulative necrosis in malignant epithelial tumors (Review)

**DOI:** 10.3892/ol.2014.2345

**Published:** 2014-07-11

**Authors:** ROSARIO A. CARUSO, GIOVANNI BRANCA, FRANCESCO FEDELE, ELEONORA IRATO, GIUSEPPE FINOCCHIARO, ANTONIO PARISI, ANTONIO IENI

**Affiliations:** 1 Department of Human Pathology, University of Messina, Messina I-98125, Italy; 2 Department of Surgical Science, University of Messina, Messina I-98125, Italy

**Keywords:** histologic tumor necrosis, mitotic catastrophe, histopathology, carcinoma

## Abstract

Histological tumor necrosis (TN) has been reported to indicate a poor prognosis for different human cancers. It is generally accepted that TN results from chronic ischemic injury due to rapid tumor growth. However, whether insufficient tumor vascularization and inadequate tumor cell oxygenation are the only factors causing TN remains controversial. Mitotic catastrophe is considered to occur as a result of dysregulated/failed mitosis, leading to cell death. We hypothesize that mitotic catastrophe, induced by hypoxic stress, may lead to the TN which is observed in high grade carcinomas. The current review describes the morphological features of TN in malignant epithelial tumors. In addition, evidence regarding the involvement of mitotic catastrophe in the induction of TN in human carcinomas is discussed.

## 1. Introduction

Recent studies have shown that tumor necrosis (TN) influences metastasis-free survival in patients exhibiting neoplasms (1,2). In particular, TN has been reported to indicate poor prognosis in lung (3), breast (4,5), thyroid (6), colorectal (7,8), pancreatic (9) and renal (10–16) malignancies. Therefore, it has been proposed that the presence/absence of TN must be indicated in any histopathological report (12,14), as this type of assessment has a high rate of reproducibility among pathologists (9,12).

It is generally accepted that TN is a result of chronic ischemic injury due to rapid tumor growth. Jain and Carmeliet (17) suggested that intratumoral mechanical stresses, resulting from tumor cell proliferation, cause focal large-vessel obstruction, leading to ischemic intratumoral infarcts. The compression, exerted by surrounding neoplastic cells on the microvasculature, is considered to be spatially and temporally heterogeneous (18) and this may explain the uneven distribution of TN. However, whether insufficient tumor vascularization and inadequate tumor cell oxygenation are the only factors causing TN remains controversial. We hypothesize that hypoxia may indirectly induce coagulative necrosis in tumor cells harboring p53 mutations via mitotic catastrophe. Mitotic catastrophe is a cell death mechanism, which occurs as a result of dysregulated/failed mitosis that may be accompanied by morphological alterations, including micronucleation, multinucleation and abnormal mitoses.

In this review, the morphologic features of TN in malignant epithelial tumors are investigated. In addition, the associations between hypoxia, mitotic catastrophe and TN are briefly reviewed within the framework of our hypothesis.

## 2. Definition of apoptosis and TN

Depending on the lethal stimulus, tumor cells may die as a result of distinct cellular death mechanisms, including apoptosis and necrosis. The term ‘apoptosis’ was coined by Kerr *et al* (19) to distinguish the phenomenon as a mechanism of cell death that is morphologically separate from coagulative necrosis. Ultrastructural features of apoptosis include margination and condensation of chromatin, nuclear fragmentation in apoptotic bodies (corresponding to the histological terms, pyknosis and karyorrhexis), and ruffling of the plasma membrane, which maintains integrity until the final stages of the process (19–21). Phagocytosis of apoptotic bodies is carried out by professional phagocytes, including macrophages and dendritic cells, and non-professional ‘neighboring’ phagocytes, including epithelial cells, endothelial cells, smooth muscle cells and fibroblasts. By contrast, necrosis is characterized by cellular swelling, which is accompanied by chromatin flocculation, dilatation of the mitochondria and endoplasmic reticulum, plasma membrane rupture and eventual shedding of the cytoplasmic contents into the extracellular space, with subsequent inflammation (20–21).

## 3. Detection of TN

According to the recommendations of the Nomenclature Committee on Cell Death (NCCD) (21–23), electron microscopy remains the ‘gold standard’ for identification of the specific features of cells undergoing death. However, the detection of cell death must be based on at least two techniques, one to reveal morphological changes and the second to demonstrate biochemical changes (21). For example, pathologists use combined immunohistochemical methods and light microscopy to identify dying necrotic cells. Histologically, coagulative necrosis appears acellular and stains homogeneously with red eosin. However, careful examination shows retention of the general architectural pattern of the tissue, despite the death of its constituent elements. Coagulative necrosis is also characterized by an abrupt transition from viable to necrotic cells without an interposed zone of granulation tissue or hyalinized tissue between the viable and necrotic cells (21). Generally, these histological observations are supplemented with electron microscopy images to identify the morphological characteristics of dying necrotic cells. In addition, Tdt-mediated dUTP nick end labeling (TUNEL) and anti-active caspase-3 staining are often used to identify apoptotic cell death (21). Usually, cells that stain positively for TUNEL but negatively for active caspase-3 are considered to be necrotic (24). On the other hand, there are no specific positive discriminative biochemical markers for the detection of necrosis *in vitro* or *in vivo*. However, it has been demonstrated that certain candidate necrotic biomarkers, including high-mobility group box 1 protein and cyclophilin A, are released by cells dying from secondary necrosis following apoptosis (24).

## 4. Morphological variants of coagulative TN

**Figure 1:**
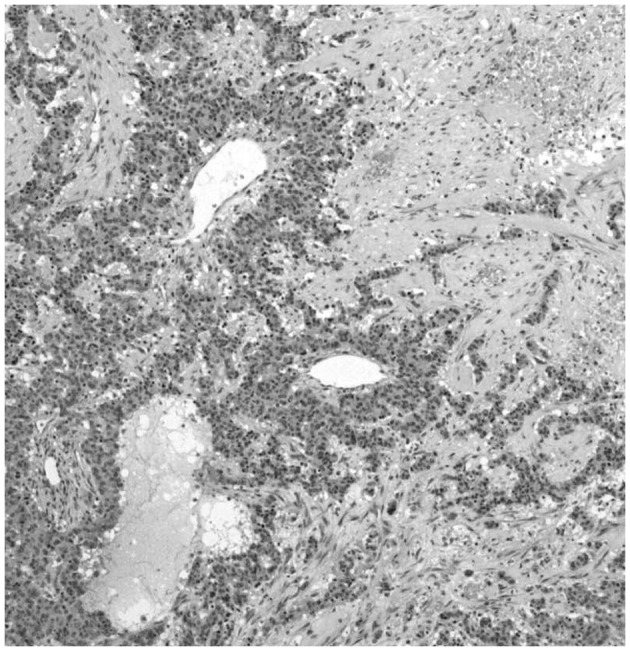
Gastric carcinoma exhibiting a solid growth pattern. Peritheliomatous necrosis is characterized by sheaths of viable tumor cells surrounding a centrally disposed blood vessel. (Stain, hematoxylin and eosin; magnification, ×100).

Peritheliomatous necrosis and comedo-type necrosis may be considered as morphological variants of coagulative TN. The term peritheliomatous necrosis refers to a microscopic pattern which is characterized by large areas of coagulative necrosis with sheets or cords of viable tumor cells surrounding a centrally disposed blood vessel (Fig. 1) (18). The term ‘comedo’ describes the appearance of compressed ducts exuding necrotic material, often observed in ductal carcinoma *in situ* (DCIS) of the breast, which is a neoplastic expansion of ductal lining cells confined by the basement membrane (25). As blood vessels remain in the stromal compartment, DCIS occurs in an avascular microenvironment and inevitably develops hypoxic regions near the oxygen diffusion limit, due to persistent proliferation of intraepithelial tumor cells. Pathologists have distinguished two types of DCIS, comedo and non-comedo (25), based on the presence of necrosis, which is often associated with microcalcifications in the center of the breast ducts (25).

A pattern similar to comedo-type necrosis, characteristically found in DCIS of the breast, has also been identified in invasive carcinomas. It is characterized by the presence of well-circumscribed epithelial nests containing central necrotic material, including neuroendocrine carcinomas; carcinoma arising in pleomorphic adenoma, duct carcinomas of the salivary glands; cervical carcinoma *in situ* with features of impending invasion; and basaloid squamous carcinoma of the lung, salivary glands, esophagus, anal canal and sinonasal tract (25). Therefore, coagulative necrosis and its variants (peritheliomatous and comedo-type necrosis) are usually observed in epithelial tumors, *in situ* and invasive, characterized by a solid growth pattern.

## 5. TN and fibrotic focus

Following a certain period of time, coagulative necrosis may be replaced by colliquative necrosis, in which the cellular structures are broken down by proteolitic enzymes released from ruptured lysosomes and similar enzymes released by infiltrating inflammatory cells (20). Finally, colliquative/coagulative necrosis is replaced by a scar-like area, defined as the fibrotic focus (26). It appears as a radially expanding fibrosclerotic core and consists of loose, dense or hyalinized collagen bundles and a variable number of fibroblasts (26). In addition, elastic tissue may be abundant. The arrangements of fibroblasts or collagen fibers forming fibrotic foci differ from that of the surrounding stroma, which is more ordered (26). The presence of a fibrotic focus was found to positively correlate with disease progression, increased tumor size, lymph node metastases and a poor outcome in breast, colorectal and pancreatic cancer (26–28).

## 6. TN in invasive adenocarcinomas

**Figure 2:**
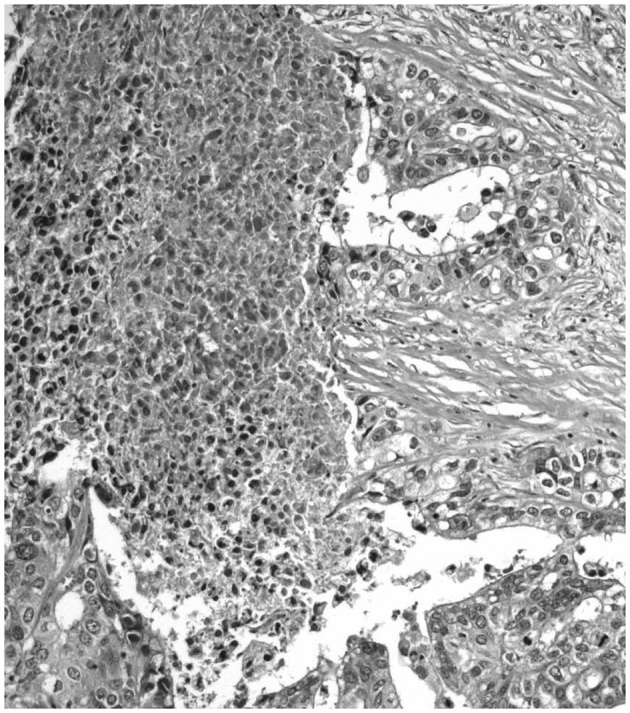
Gastric adenocarcinoma: Necrotic areas involving the stroma and epithelium are infiltrated by neutrophils. (Stain, hematoxylin and eosin; magnification, ×100).

Colliquative necrosis, dirty necrosis and intraglandular necrotic debris are usually identified in invasive adenocarcinomas (29–35). In these tumors, necrosis may remain confined to single neoplastic glands, whereas in other areas it may involve neoplastic glands and intervening stroma. The term ‘dirty necrosis’ is used to describe the presence of intraglandular eosinophilic material frequently in combination with necrotic cell debris and neutrophils (29). This intraglandural material stains positively with periodic acid-Schiff and expresses the transmembrane glycoprotein MUC1 (29). Furthermore, dirty necrosis has been identified in colorectal adenocarcinomas and is often accompanied by segmental necrosis of the glandular lining (29). Foci of dirty necrosis are also common in pulmonary metastases of colonic carcinomas, but are rarely observed in primary lung adenocarcinomas (36). Necrotic areas involving the stroma and glands are frequently infiltrated by neutrophils in a pattern similar to that observed in colliquative necrosis (Fig. 2) (7). Notably, mucinous adenocarcinomas are characterized by MUC2 overexpression and the absence of dirty necrosis (37). Colliquative and/or dirty necrosis are predominantly found in MUC1-positive adenocarcinomas of the pancreas (38) and colorectum (29), whereas the absence of necrotic phenomena is characteristically found in MUC2-positive mucinous adenocarcinomas of the gastrointestinal tract (36,39).

## 7. p53

**Figure 3:**
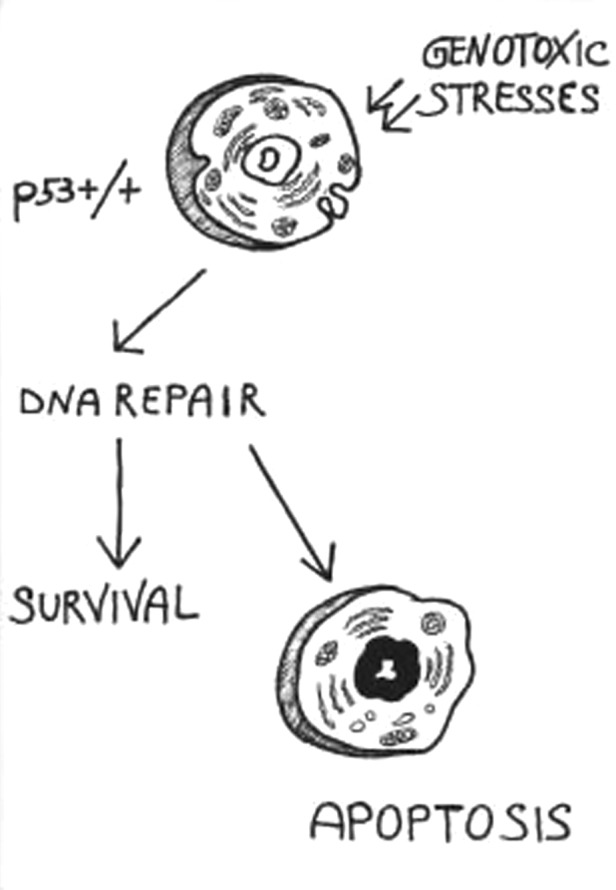
Schematic representation of the pathway leading to apoptosis in wild-type p53 (+/+) cells following genotoxic stress.

p53 acts as a guardian of the genome, protecting cells against cancer (40). In response to a variety of genotoxic stresses (DNA-damaging agents, UV damage, antimicrotubule agents and hypoxia), the p53 protein promotes cell-cycle arrest, which is necessary to repair any DNA damage, or apoptosis, if repair cannot be achieved (Fig. 3) (40). Cell-cycle arrest may be used to repair any damage, whereas apoptosis is a genetically controlled response whereby cells commit suicide when repair cannot be achieved. These cellular responses allow p53 to inhibit tumorigenesis and genomic instability (40). Furthermore, when p53 is mutated, it accumulates at the nuclear level and the cell-cycle checkpoint becomes defective. Thus, a cell may enter mitosis prematurely, prior to the completion of DNA replication or DNA damage repair. This aberrant mitosis may lead to apoptosis or necrosis (41). Of note, mitotic catastrophe is not considered a form of cell death, but rather an irreversible trigger for cell death (22).

**Figure 4:**
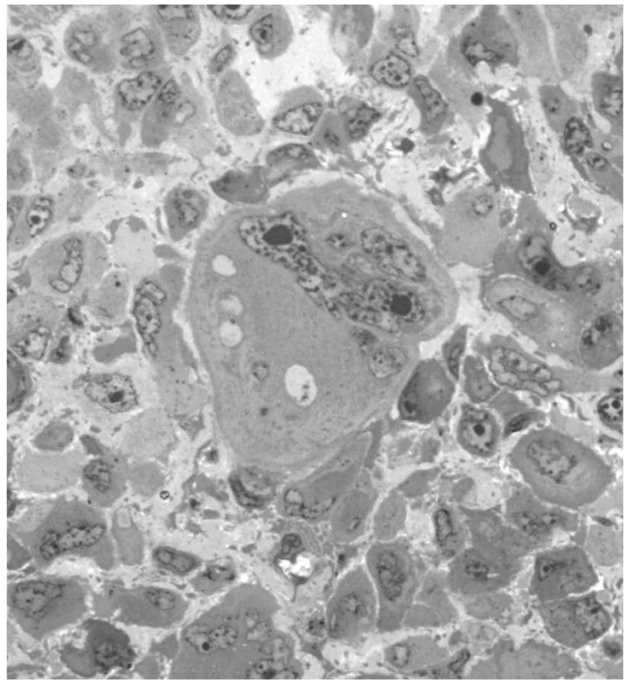
Semi-thin section of pleomorphic giant cell carcinoma of the thyroid. Certain giant tumor cells exhibit multiple nuclei and micronuclei. (Stain, Giemsa; magnification, ×400).

The p53 tumor suppressor gene is mutated in ~50% of all human cancers. Following severe genotoxic damage, numerous p53-mutated tumors undergo mitotic catastrophe (41–44). According to NCCD, mitotic catastrophe refers to cell death that is triggered by aberrant mitosis and executed during mitosis or in the subsequent interphase (22). Mitotic catastrophe is morphologically characterized by anisocytosis and anisokaryosis (heterogeneity in cytoplasmic and nuclear size, respectively), presence of micronuclei (derived from chromosomes and/or chromosome fragments that have been irregularly distributed between daughter nuclei) and multinucleation (two or more nuclei with similar or heterogeneous sizes in a single cell, as a result of failed separation during cytokinesis) (Fig. 4) (22). Morphological features associated with mitotic catastrophe may be observed in pleomorphic, giant cell carcinoma, a tumor without any identifiable glandular, squamous or any other type of differentiation (45). It consists of sheets of highly undifferentiated pleomorphic cells, often with areas of coagulative necrosis (22) with numerous bizarre/multinucleated cells (46–48) and many abnormal mitotic figures. Pleomorphic, giant cell carcinomas are highly malignant tumors that are most commonly found in the lungs, breast, pancreas and thyroid (22).

## 8. Mitotic catastrophe in anticancer therapy

Mitotic catastrophe has been characterized as the predominant form of cell death induced by ionizing radiation, and occurs in response to several anticancer drugs (49,50). Since preoperative chemotherapy is being used more frequently in the management of advanced tumors, pathologists must be aware of the resultant morphological effects, which may result in difficulties in tumor typing and grading and in the identification of residual neoplasia (51,52). The morphological features of lung, breast and ovarian cancers treated with chemotherapy include nuclear and cytoplasmic alterations and pronounced stromal changes (52,53). Nuclei exhibit significant enlargement with extremely irregular outlines, and occasionally appear similar to multinucleated giant cells (52,53), a feature associated with mitotic catastrophe. Nuclear size has been shown to represent a useful prognostic indicator in ovarian and breast cancer, and therefore an increased nuclear size post-chemotherapy may influence the results if this measurement is used as a predictor of outcome (53). Post-chemotherapy tumor cells are observed singularly or in small clusters, often without tubular differentiation, and mitotic activity is rare (53). Therefore, preoperative chemotherapy causes difficulty in tumor grading, which is based on cytological and architectural features, as well as mitotic activity.

## 9. Pathogenesis of TN

**Figure 5:**
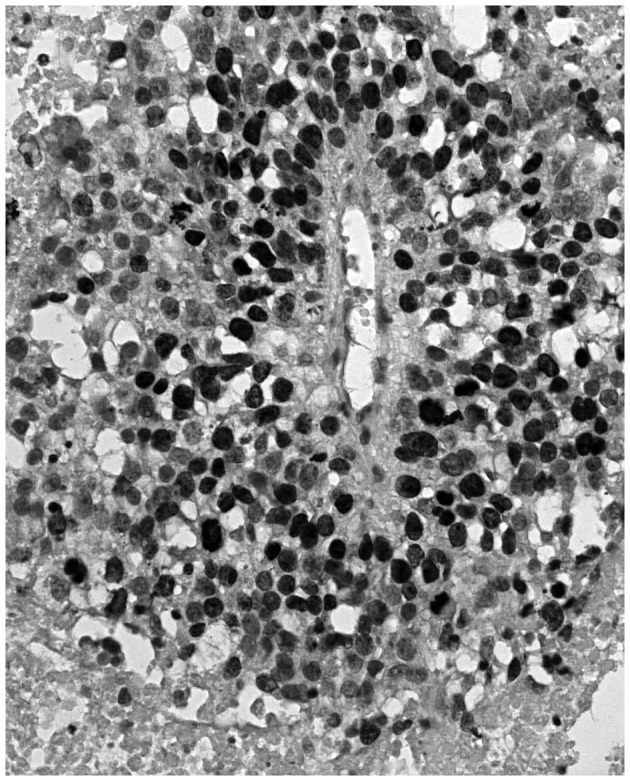
Gastric carcinoma: A close association between peritheliomatous necrosis, atypical mitoses and numerous cycling Ki-67-positive tumor cells is observed (stain, Ki-67 immunoperoxidase; magnification, ×200).

It is hypothesized that TN is caused by chronic ischemia (i.e. hypoxia, low pH, low glucose and high lactate) within tumors, due to vascular collapse, high interstitial pressure and/or rapid tumor growth exceeding its blood supply. Anemia, the most common cancer-associated morbidity, further reduces the blood capacity for O_2_ transportation (54), and it is an adverse prognostic factor for survival, which is independent of tumor type (55). The contiguous or sheet-like nature of the necrosis indicates that the cause of death is due to ischemic injury, affecting a field or group of tumor cells, supplied or drained by a single vessel (18). The subsequent necrosis suggests that the large feeding artery or exit vein becomes obstructed, leading to an arterial or a venous infarct (18). By contrast to this hypothesis, it has been revealed that TN frequently occurs within regions that display relatively increased microvessel density (56). However, there are tumors in which coagulative necrosis is rare, although the tumor stage is advanced. For example, the lowest frequency of TN is observed in mucinous adenocarcinomas of the gastrointestinal tract (36). Furthermore, Tollefson *et al* (57) revealed that renal carcinomas exhibiting coagulative necrosis also exhibited relatively high proportions of proliferative Ki-67-positive tumor cells. Similar findings have been demonstrated in gastric carcinomas (45). Fig. 5 shows the morphological association between peritheliomatous necrosis, atypical mitoses and high proportion of cycling Ki-67 immunoreactive tumor cells.

**Figure 6:**
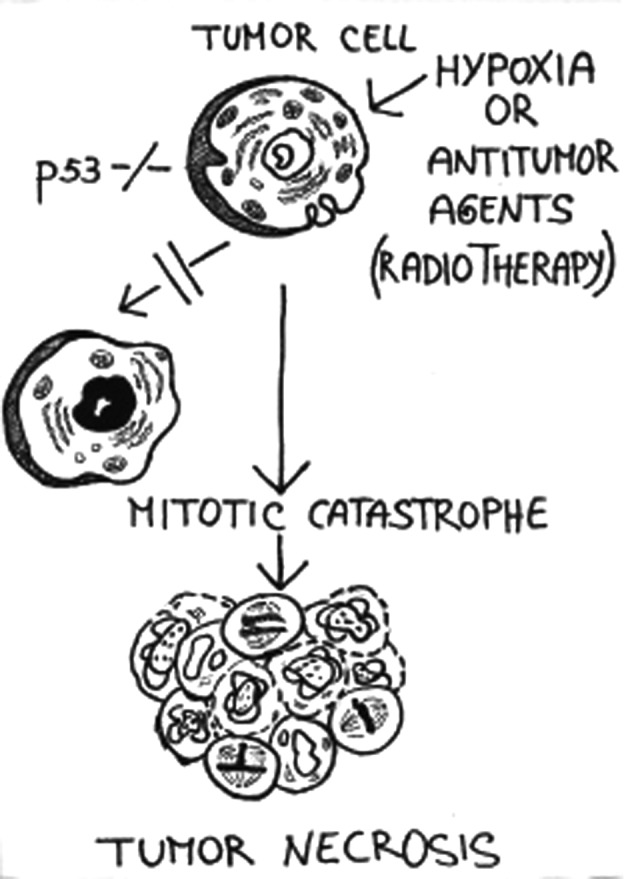
Proposed model of tumor necrosis in mutated p53 (−/−) tumor cells. Ischemia, a known genotoxic stress, may induce necrosis via mitotic catastrophe variably accompanied by atypical mitoses, anisocytosis, anisokaryosis, multinucleation and micronucleation.

We hypothesize that hypoxia, a known genotoxic factor, may indirectly induce TN via mitotic catastrophe in tumor cells harboring p53 mutations. A similar pathway has been suggested for TN occurring *in vivo* following treatment with anticancer drugs or radiation (42,44,58). Our hypothesis for the association between hypoxia, mitotic catastrophe and TN is shown in Fig. 6. Further studies regarding the mechanisms associated with TN may yield useful insights into epithelial malignant tumor biology and improve patient management.
